# Length of postoperative stay prediction in elderly patients with hip fractures based on machine learning

**DOI:** 10.3389/fmed.2025.1728645

**Published:** 2026-01-14

**Authors:** Yanli Hu, Hong Qu, Feifan Wang, Fangfang Deng, Qun Luo, Tingting Gong

**Affiliations:** 1 Department of Orthopedics, Yichang Central People’s Hospital, Yichang, China; 2 College of Medicine and Health Science, China Three Gorges University, Yichang, China; 3 Department of Patient Services, Yichang Central People’s Hospital, Yichang, China; 4 Department of Outpatient, Yichang Central People’s Hospital, Yichang, China; 5 Department of Critical Care, Yichang Central People’s Hospital, Yichang, China

**Keywords:** BP neural network, elderly, hip fractures, length of postoperative stay, length of stay, machine learning, predict

## Abstract

**Background:**

Length of postoperative stay (LOPS) is an important indicator for resource allocation and clinical management in elderly patients with hip fractures. However, previous studies have mostly dichotomized this continuous variable to determine whether it is prolonged, a practice that inherently reduces information and introduces limitations. This study aimed to develop and validate a machine learning (ML) model to accurately predict the specific LOPS in elderly patients with hip fractures.

**Methods:**

This retrospective cohort study included electronic health records (EHRs) of elderly patients with hip fractures admitted to Yichang Central People’s Hospital from January 2016 to December 2022, with a total of 734 patients. Variables commonly measured preoperatively were extracted based on a review of previous studies, and features were selected using Pearson correlation coefficients combined with LASSO regression to construct a backpropagation neural network (BP-NN) model. For comparative evaluation, support vector machine (SVM) and random forest (RF) regression models were developed under the same dataset split (8:2), feature set, and hyperparameter optimization strategy. Model performance was assessed by comparing predicted values versus actual LOPS and calculating root mean square error (RMSE), mean absolute error (MAE), mean absolute percentage error (MAPE), and error thresholds (20%, 30%). The feature importance of the BP-NN model was analyzed via SHapley Additive exPlanations (SHAP) values.

**Results:**

Among 734 elderly patients with hip fractures, 503 (68.53%) were female, with an average LOPS of 17.42_±_ 3.77 days. Femoral neck fracture (59.26%) and hemiarthroplasty (41.96%) were the most common fracture type and surgical type, respectively. Pearson correlation analysis and LASSO regression showed that age, age-adjusted Charlson comorbidity index (ACCI), and surgical type were the predictors of LOPS. Further sensitivity analysis adjusting for confounding factors revealed that the very old elderly group (aged or above 90 years) had the longest LOPS (15.84_±_ 0.15 days vs. 17.85_±_ 0.14 days vs. 21.99 _±_ 0.66 days), with no statistically significant difference in LOPS between different surgical type subgroup (*P* > 0.05). The predicted values of the BP-NN were consistent with the trend of actual LOPS (*R*^2^ = 0.83), with the vast majority of prediction results falling within 30% clinically acceptable error threshold. Its RMSE, MAE and MAPE of 1.23 days, 1.57 days and 7.69% respectively. SHAP analysis revealed that ACCI and age were the main factors influencing LOPS.

**Conclusion:**

The BP-NN model, enhanced by multimethod feature selection, rigorous parameter tuning, and SHAP based interpretability, provides early and accurate LOPS prediction for elderly hip fracture patients. It can be used as a tool to assist in clinical decision-making, resource planning, and discharge preparation, without increasing the clinical burden. Future external validation across multiple centers is needed to confirm generalizability.

## 1 Introduction

Hip fractures, highly prevalent among the elderly, are characterized by poor prognosis, frequent complications, and elevated mortality rates (1–3). Length of postoperative stay (LOPS) serves as a critical indicator of clinical outcomes and nursing quality, objectively reflecting healthcare efficacy for elderly patients with hip fracture (4). The unplanned prolongation of LOPS may lead to an increased risk of complications in patients and higher medical expenses (5–7). Previous studies have revealed that each additional day of hospitalization increases complications risk by 5%, and hospitalization costs increase by 5–8% accordingly (8, 9). In addition, the unplanned shortening of LOPS may adversely affect postoperative rehabilitation of patients, leading to an increased risk of complications or even death (10, 11). Therefore, accurately predicting the specific LOPS in elderly patients with hip fracture is crucial for healthcare institutions and patients.

Prior retrospective studies (12–16) have mostly used classification methods to explore LOPS distribution in elderly patients with hip fractures, mainly focusing on whether LOPS is prolonged. However, artificially classifying a continuous variable like LOPS essentially involves the subjective assumption that there are inherent differences between subjects below and above the threshold. This practice may introduce bias and information loss, leading to unreliable results (17, 18). Therefore, establishing a statistical model with LOPS as a continuous variable for regression analysis can fully preserve the information on individual differences, allowing for more precise quantification of the association between each influencing factor and LOPS. Currently, the revelated study predicting LOPS as a continuous variable has mostly focused on patients undergoing total hip arthroplasty (THA) (19). Thus, constructing a model capable of accurately predicting LOPS in all elderly patients with hip fractures will be more conducive to ward risk management and the allocation of precise medical services.

Machine learning (ML) algorithms, such as neural networks, can better handle nonlinear relationships between variables and complex interactions among multiple factors, which is particularly critical for LOPS prediction as it is influenced by multiple interconnected clinical factors (20). Compared with traditional statistical methods, ML is more capable of revealing the interactive effects and underlying associations among multiple factors, and has been widely applied in the field of medical outcome prediction with excellent performance (21, 22). Meanwhile, neural network is the most commonly used algorithm for time-related outcome prediction (23), further supporting the feasibility of this approach. Therefore, this study aimed to develop and validate a neural network for predicting the specific LOPS in elderly patients with hip fractures.

## 2 Materials and methods

### 2.1 Study design and patient population

This study was approved by the Hospital Ethics Committee of Yichang Central People’s Hospital (Approved No.: 2024-138-01). This retrospective study initially screened electronic health records (EHRs) data of all elderly patients (aged 65 years and above) with hip fractures from Yichang Central People’s Hospital, China. Between January 2016 and December 2022, a total of 775 elderly patients with hip fractures underwent THA, hemiarthroplasty (HA), or internal fixation (IF). These three procedures are the most commonly used treatment methods for elderly patients with hip fractures (24), ensuring the study population’s representativeness and the model’s clinical utility. Among the 775 patients, 41 cases were excluded: (1) postoperative transfer to another hospital (25 cases, incomplete medical records unfit for analysis); (2) postoperative hospital stay exceeding 60 days (3 cases, inconsistent with the study’s focus on routine stays); (3) in-hospital death (n = 13). Eventually, a total of 734 cases were utilized to develop ML algorithms for predicting the specific LOPS of elderly patients with hip fractures.

### 2.2 Primary outcome

The primary outcome was to predict the specific LOPS in elderly patients with hip fractures. LOPS was defined as the number of days from the day after surgery to the day of discharge (25). The secondary outcome of interest was to identify the preoperative factors influencing LOPS in elderly patients with hip fractures.

### 2.3 Feature selection

Candidate variables were selected based on established associations with LOPS in prior studies (12, 13, 16), included age, sex, age-adjusted Charlson comorbidity index (ACCI), fracture types, and surgical type. For elaboration, ACCI was developed from the Charlson Comorbidity Index, which incorporates age-related prognostic impacts with stage-specific weighting (26). Comorbidities included coronary atherosclerotic heart disease, peripheral vascular disease, heart failure, stroke, limb paralysis, peptic ulcer, liver disease, chronic obstructive pulmonary disease, diabetes, chronic kidney disease, solid tumors, and hematological malignancies (lymphoma, leukemia) to quantify overall health risks.

All candidate variables were common clinical indicators, so there were no missing data. Continuous variables were subjected to mean normalization to eliminate the influence of different measurement scales on model training, and categorical variables were one-hot encoded to convert nominal data into numerical format suitable for model input. This study employed Pearson correlation coefficient analysis and LASSO regression to quantify the association between each candidate variable and LOPS, while enhancing accuracy and reducing computation time. Pearson was used to analyze the correlations both between individual variables and between individual variables and the LOPS label, aiming to identify and eliminate multicollinearity among variables (27). The optimal regularization parameter of LASSO regression was determined via 10-fold cross-validation. Variables with non-zero coefficients after regularization were retained for subsequent construction of the LOPS predictive model.

### 2.4 Machine learning development and validation

The study cohort was divided into a training dataset and an independent validation dataset using an 8:2 stratified split ratio according to previous studies (28, 29). To reduce potential bias from a single split, we further performed 10-fold cross-validation on the training dataset to evaluate the model’s stability. Based on the feature selection results, this study adopted the backpropagation neural network (BP-NN) as the core predictive model and trained it on the training dataset. As a typical multi-layer feedforward neural network, the BP-NN consists of an input layer, one or more hidden layers, and an output layer. It minimizes prediction errors by backpropagating output errors, calculating gradients of each layer, and iteratively adjusting connection weights (30). The number of neurons in the input layer is consistent with the dimensionality of the model’s input features to ensure the complete input of original feature information, while the number of neurons in the output layer is set corresponding to the dimensionality of the predicted variables to match the task’s output requirements. Considering the limited number of study samples, a single hidden layer was configured in the network architecture to mitigate overfitting risk. At present, there is no standardized method for determining the number of hidden layer neurons (31), and this number is commonly derived from the neuron counts of the input and output layers. This study selected the tansig activation function for the hidden layer and the purelin function for the output layer. The tansig function was used to model nonlinear relationships in medical data and capture complex nonlinear interactions between variables- a capability critical for LOPS prediction involving multiple clinical factors (31). The purelin function enabled linear output mapping, which was well-suited to the continuous nature of LOPS values.

In addition, two machine learning models, support vector machine (SVM) and random forest (RF), were constructed under the same dataset and hyperparameter optimization conditions to conduct model performance comparison. These models were chosen as a comparator due to their prior performance in continuous variable prediction and widespread use for surgical outcomes prediction (29, 32). To ensure model performance and effective data feature capture, we adopt grid search combined with 10-fold cross-validation for hyperparameter optimization on the training dataset, and the configuration corresponding to the minimum root mean square error (RMSE) was selected for each model (BP-NN: learning rate, number of hidden layer neurons, additional momentum factor, number of training iterations; SVM: kernel function type, penalty coefficient, kernel function parameter; RF: number of decision trees, maximum tree depth, minimum number of samples per leaf node). To prevent overfitting, we introduced early stopping during training, which is a simple and efficient regularization technique that monitors validation performance trends and terminates training before the model shows overfitting signs. The performance of the prediction models was comprehensively evaluated by comparing the actual LOPS with predicted values in the validation dataset, using metrics including RMSE, mean absolute error (MAE) and mean absolute percentage error (MAPE). R^2^ was also calculated to evaluate the goodness of fit of the model. SHapley Additive exPlanations (SHAP) values were computed to interpret the BP-NN model, which could quantify the contribution of each feature to the prediction result and enhanced the clinical interpretability of the model.

### 2.5 Statistical analysis

This study was performed using MATLAB software (Windows, Version: 2023b) for data preprocessing and statistical analysis. Categorical variables were presented as counts (percentages) and comparisons between groups were conducted using the chi-square test or Fisher’s exact test. Continuous variables were first assessed for normality via the Shapiro-Wilk test. Those following a normal distribution were described as the mean with the addition and subtraction of the standard deviation (SD), non-normally distributed variables as median (interquartile range), and compared using the Mann-Whitney U test.

## 3 Results

### 3.1 Study population

This study included 734 elderly patients with hip fractures: 587 in the training dataset and 147 in the validation dataset. Of these patients, 503 were women (68.53%), and the average LOPS was 17.42_±_ 3.77 days. The most common fracture types were femoral neck fracture (59.26%), with HA (41.96%) as the most frequent surgical type. To avoid bias in the results caused by the uneven distribution of the dependent variable, we compared the baseline characteristics of elderly patients with hip fractures between the training dataset and validation dataset Table 1. The results showed no significant differences in clinical characteristics between the two groups (*p*> 0.05).

**TABLE 1:** Baseline characteristics of study population.

Characteristics	Training dataset (587 cases)	Validation dataset (147 cases)	*P*-value
Sex [n, (%)]			0.80
Male	186(31.69)	45(30.61)	
Female	401(68.31)	102(69.39)	
Age (years, $\bar{x}$ ± s)	76.93 ± 6.98	78.19 ± 7.23	0.07
ACCI (scores, $\bar{x}$ ± s)	3.95 ± 1.20	4.02 ± 1.09	0.53
Fracture types [n, (%)]			0.11
Femoral neck fractures	359(61.16)	76(51.70)	
Intertrochanteric fractures	216(36.80)	68(46.26)	
Subtrochanteric fractures	12(2.04)	3(2.04)	
Surgical type [n, (%)]			0.14
THA	138(23.51)	24(16.33)	
HA	245(41.74)	63(42.86)	
IF	204(35.75)	60(40.82)	
LOPS (days, $\bar{x}$ ± s)	17.32 ± 3.75	17.96 ± 3.91	0.07

### 3.2 Feature selection

**FIGURE 1:**
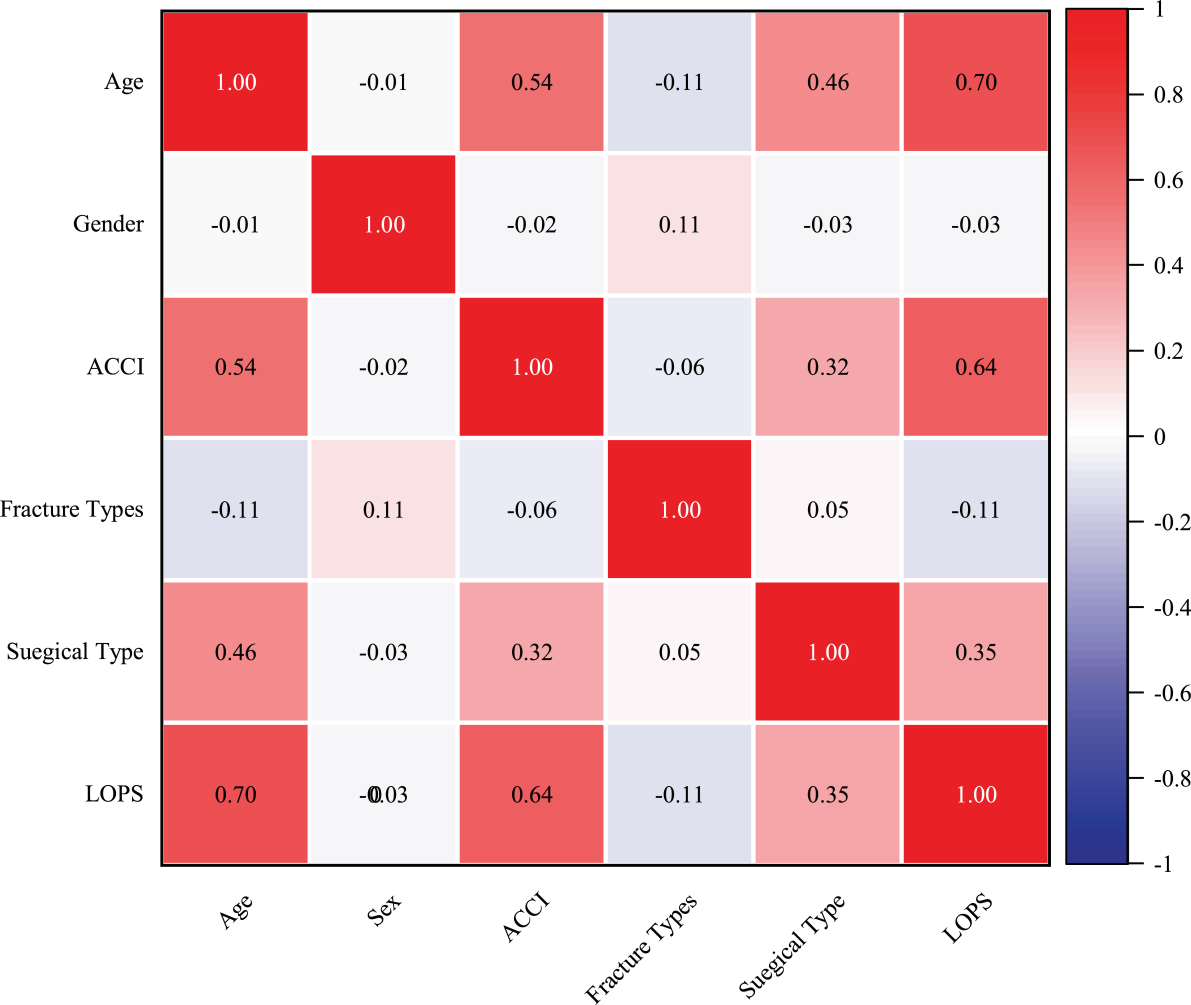
Correlation heatmap.

**FIGURE 2:**
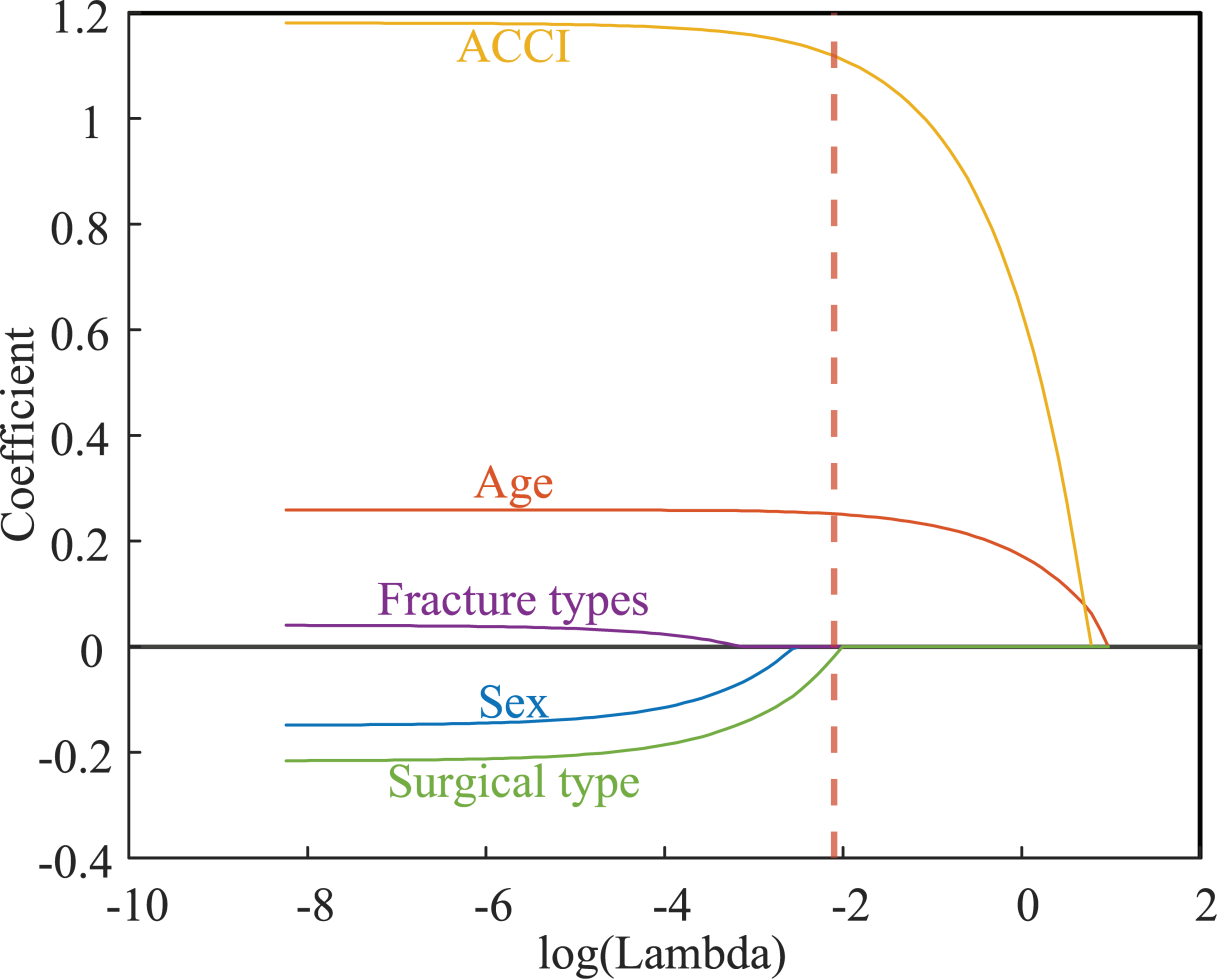
Feature selection using LASSO regression.

Pearson correlation analysis revealed that age, and ACCI were strongly correlated with LOPS, while surgical type was weakly correlated (Figure 1). Subsequently, LASSO regression confirmed that age, ACCI and surgical type were the predictors for LOPS in this patient population (Figure 2).

Based on the above feature selection results, we further explored the association between the different age groups and three surgical types with LOPS. Age groups were categorized in accordance with the latest elderly age stratification criteria of the World Health Organization (WHO): 60–74 years as the young elderly group, 75–89 years as the older elderly group, and aged or above 90 years as the very old elderly group. The relevant results were presented in Tables 2, 3.

**TABLE 2:** Baseline comparison among elderly patients with hip fractures of all age groups.

Characteristics	Young elderly group (*n* = 287)	Older elderly group (*n* = 423)	Very old elderly group (*n* = 24)
ACCI (scores, $\bar{x}$ ± s)	3.31 ± 1.07	4.36 ± 1.05	4.88 ± 0.74
THA [n, (%)]	123(42.86)	39(9.22)	0(0)
HA [n, (%)]	61(21.25)	229(54.14)	18(75.00)
IF [n, (%)]	103(35.89)	155(36.64)	6(25.00)
LOPS (days, $\bar{x}$ ± s)	14.95 ± 2.15	18.78 ± 3.58	24.10 ± 2.65
Adjusted LOPS (days, $\bar{x}\pm s$)	15.84 ± 0.15	17.85 ± 0.14	21.99 ± 0.66

**TABLE 3:** Baseline comparison of three surgical types for elderly patients with hip fractures.

Characteristics	THA (*n* = 162)	HA (*n* = 308)	IF (*n* = 264)
Age (years, $\bar{x}$ ± s)	71.41 ± 4.32	80.18 ± 6.44	77.22 ± 7.19
ACCI (scores, $\bar{x}$ ± s)	3.27 ± 1.10	4.31 ± 1.06	4.00 ± 1.17
LOPS (days, $\bar{x}$ ± s)	14.91 ± 2.14	18.56 ± 3.79	17.70 ± 3.86
Adjusted LOPS (days, $\bar{x}$ ± s)	17.80 ± 0.28	18.71 ± 0.24	18.67 ± 0.22

Table 2 showed obvious numerical differences in LOPS across different age groups. The very old elderly group had the longest LOPS both before and after adjusting for confounding factors. Meanwhile, the comorbidity burden of elderly patients with hip fractures (as measured by ACCI score) demonstrated an increasing trend with advancing age. Distinct intergroup differences were observed in surgical type distribution: the young elderly group mainly received THA (42.86%), while HA dominated in the older elderly group (54.14%) and very old elderly group (75.00%).

Table 3 revealed that without controlling for confounding factors, there were significant differences in LOPS among the three surgical types. However, after adjusting for age and ACCI, no statistically significant independent effect of surgical type on LOPS was observed (*F* = 2.454, *p* = 0.087). This indicated that the impact of surgical type was not isolated but was conditional on patient-specific factors. BP-NN, as a universal function approximator, possesses the inherent capability to automatically discover and model such complex, non-linear interactions from the data (33). Therefore, we retained surgery type (converting THA, HA, and IF into a single categorical variable through categorical coding) together with age and ACCI as input features to construct a prediction model for LOPS in elderly patients with hip fractures.

### 3.3 Model development

Based on the identified key predictors, the number of input layer neurons in the BP-NN model was set to 3, and the number of output layer neurons was 1. The RMSE values for model with different numbers of hidden layer neurons were calculated via 10-fold cross-validation (Table 4).

**TABLE 4:** Screening of hidden layer nodes.

The number of hidden layer neurons	Average RMSE
3	1.3170
4	1.2346
5	1.7942
6	1.5649
7	1.4460
8	1.4673
9	1.7162
10	1.7312
11	1.7364
12	1.7726

**FIGURE 3:**
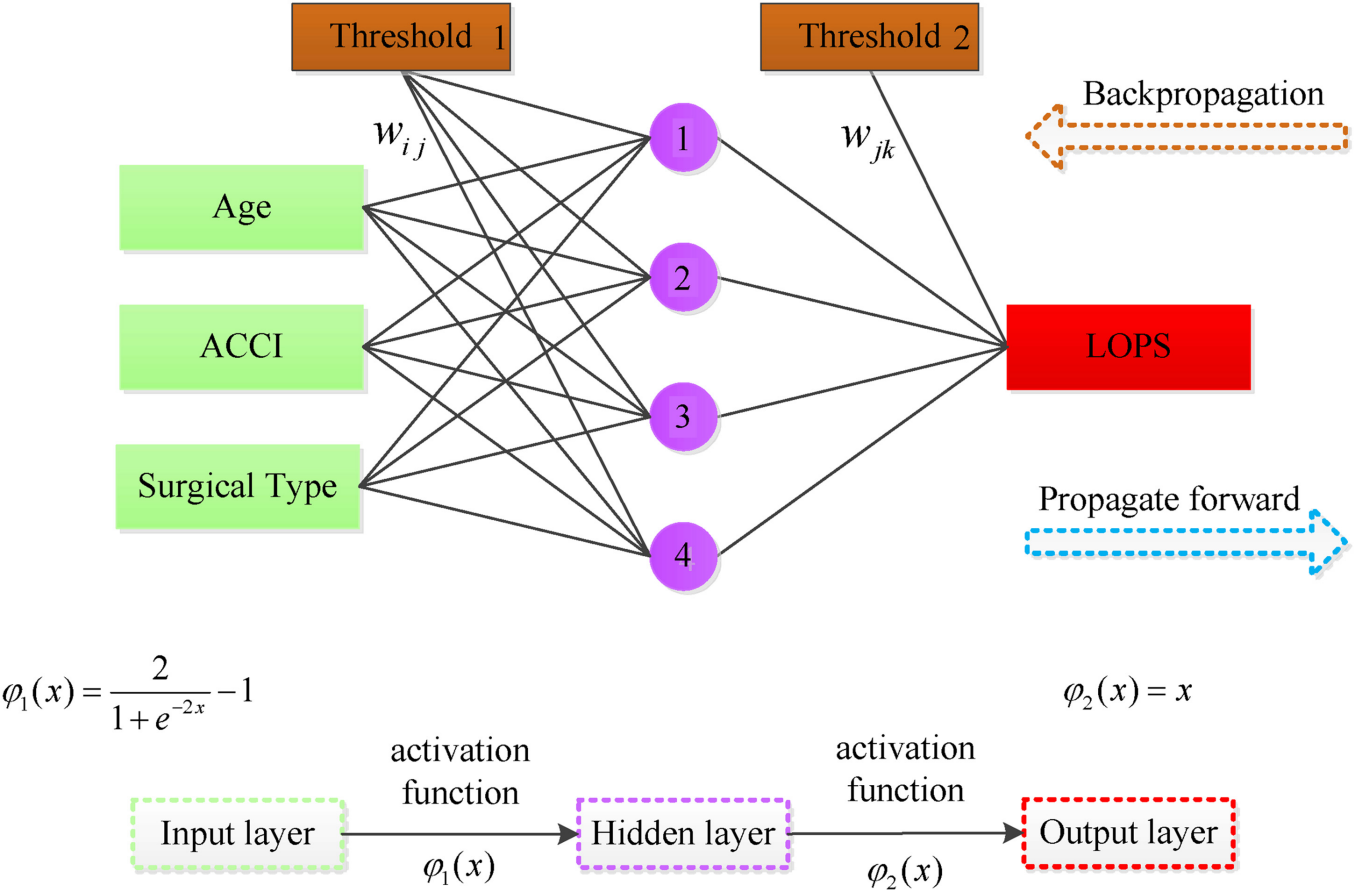
Topology of the BP-NN.

The screening results for hidden layer nodes showed that the model achieved the minimum RMSE (1.2346) with 4 hidden neurons, indicating the highest prediction accuracy. The BP-NN constructed accordingly had a topological structure of 3-4-1, as illustrated in Figure 3.

### 3.4 Model validation

**FIGURE 4:**
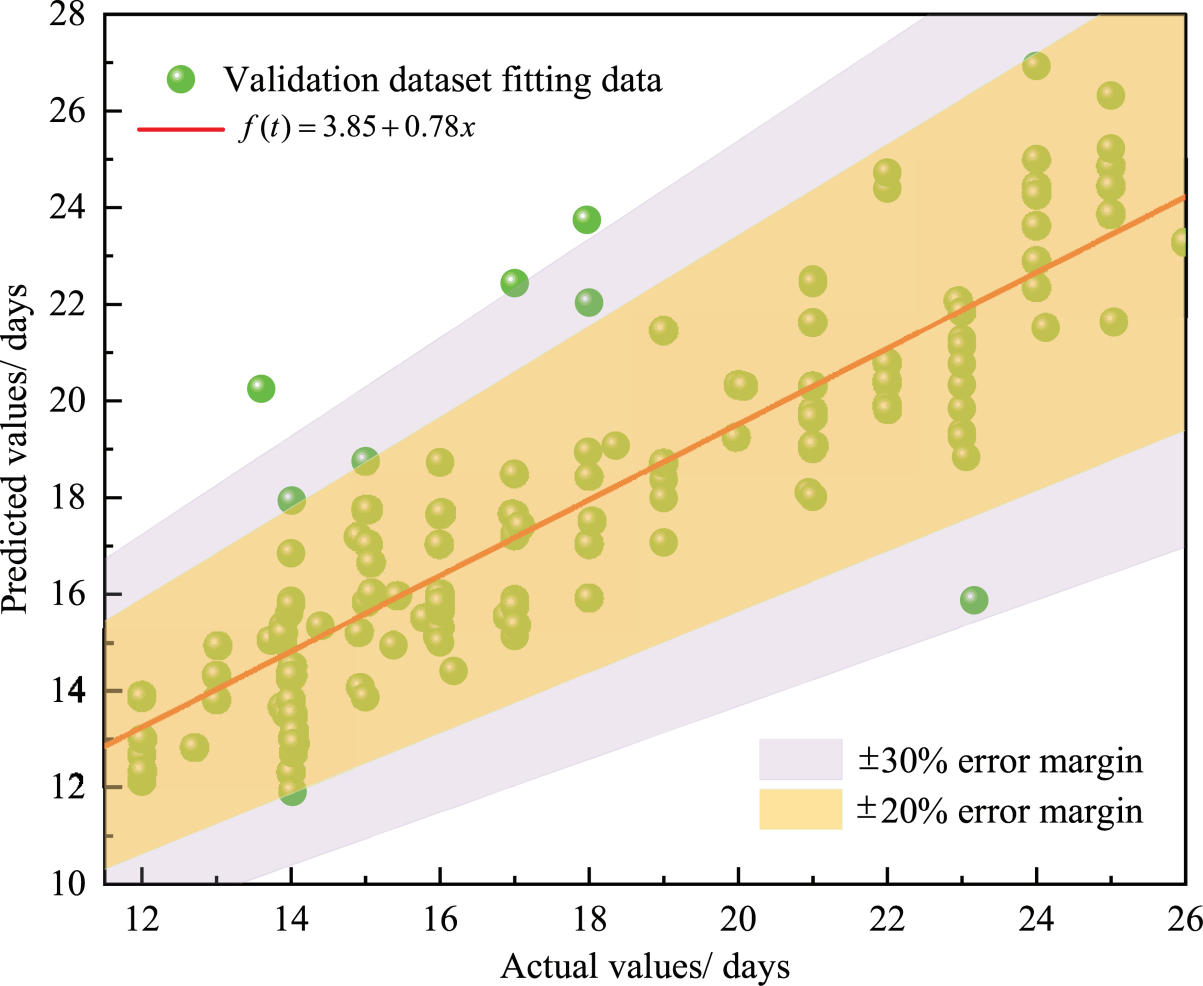
The fitting plot of predicted values and actual LOPS in the validation dataset.

To evaluate the predictive performance of the model, statistical metrics were calculated on the training dataset and the validation dataset (Table 5), and the results indicated that the model had no significant overfitting. Furthermore, a clear fitting plot of predicted values and actual LOPS in the validation dataset (Figure 4) showed that most data points were concentrated within the ± 20% error margin, with only a small number of points falling into the ± 30% error margin. Meanwhile, the data points generally exhibited a positive correlation trend along the fitted line, which indicated that the BP-NN model’s predicted values had good consistency with the actual LOPS.

**TABLE 5:** Comparison of the performance of BP-NN model.

Statistical metric	Training dataset	Validation dataset
RMSE (days)	1.15	1.23
MAE (days)	1.81	1.57
MAPE (%)	8.60	7.69

The results of the prediction model constructed using the same training dataset and hyperparameter optimization methods were shown in Table 6. The BP-NN model exhibited superior fitting performance on the validation dataset (*R*^2^ = 0.83), outperforming SVM (*R*^2^ = 0.57) and RF (*R*^2^ = 0.60). Compared to SVM, BP-NN also had lower error metrics, with RMSE being decreased by 1.33 days, MAE by 0.3 days, and MAPE by 2.68%. Compared to RF, BP-NN’s RMSE was decreased by 1.24 days, MAE by 0.25 days, and MAPE by 2.44%. These results indicated that the BP-NN model achieved favorable performance in predicting LOPS in elderly patients with hip fractures.

**TABLE 6:** Comparison of the performance of machine learning models.

Statistical metric	BP-NN	SVM	RF
RMSE (days)	1.23	2.56	2.47
MAE (days)	1.57	1.87	1.82
MAPE (%)	7.69	10.37	10.13
*R* ^2^	0.83	0.57	0.60

### 3.5 The importance of features

**FIGURE 5:**
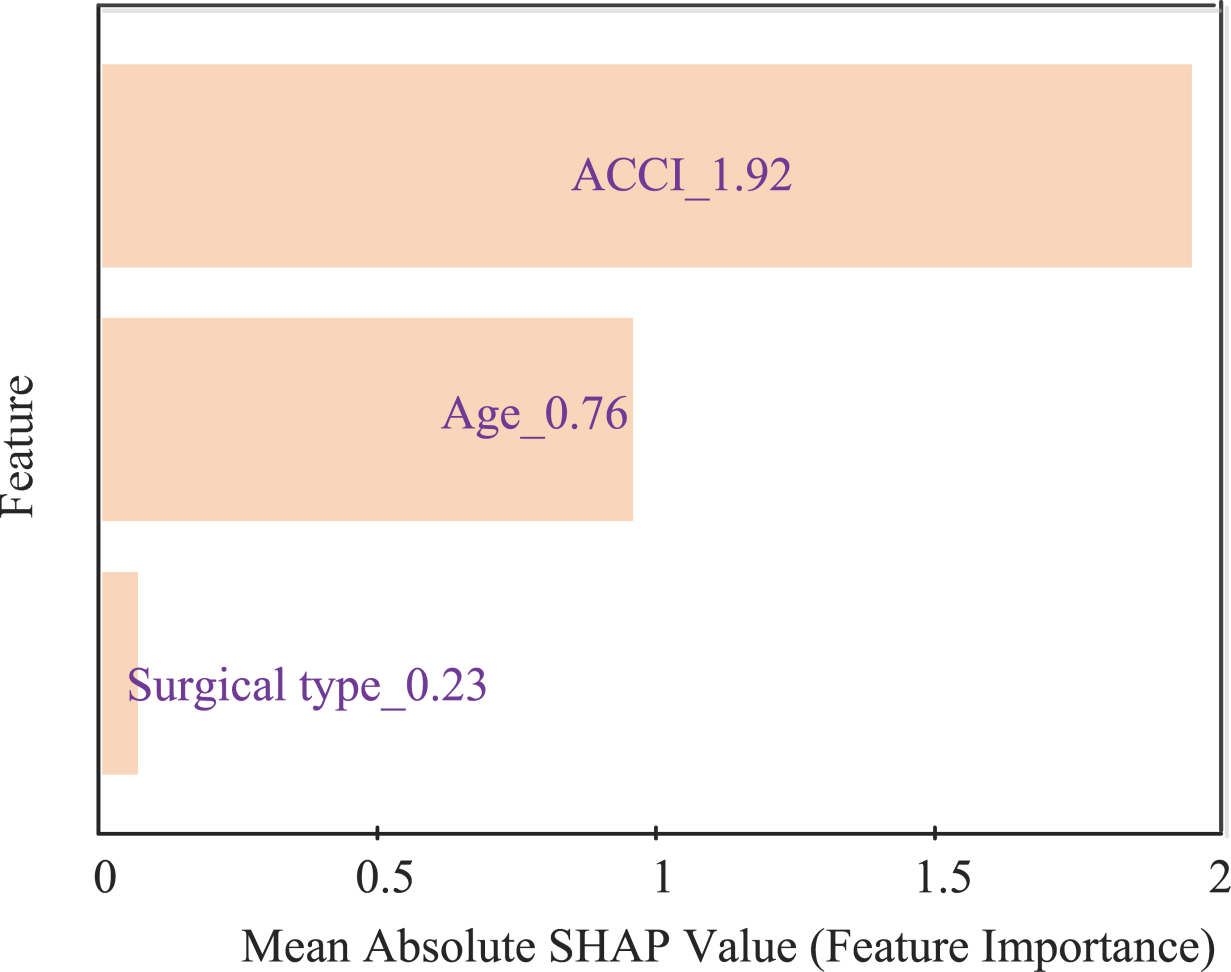
SHAP feature importance bar plot.

SHAP analysis was used to interpret the BP-NN model (Figure 5). The SHAP feature importance bar plot showed that ACCI and age were the main factors influencing LOPS, and the surgical type was secondary influencing factors of LOPS.

## 4 Discussion

This retrospective cohort study developed and validated a ML model based on EHRs of elderly patients with hip fractures to predict the specific LOPS. We confirmed age and ACCI as dominant predictors, and impact of surgical type was mediated through interactions with age and ACCI. The goal of this model was to provide medical staff with a reference for the specific LOPS through accurate calculation of continuous variables, enabling clinicians to anticipate clinical risks and support precise resource allocation in hospital management. Most importantly, the model only adopted three commonly available clinical variables, which would not increase the workload of medical staff or medical costs, making it easier to implement in hospital settings. Additionally, all variables included in the model could be determined at the early stage of admission, enabling early planning for clinical surgeons and hospital managers.

In a retrospective study collecting clinical data of patients who underwent THA from January 2014 to December 2019, the authors reported that for each additional day of hospital stay, the risk of complications in patients increased by 13% (34). However, LOPS reduction was mainly achieved through the rational allocation of medical service providers and medical resources based on conditions of patients and treatment needs, with the key being the accurate identification of the actual LOPS required by patients. Previous studies had linked risk factors to the LOPS through linear regression analysis (12, 13, 16), but most of these adopted threshold-based classification, focusing on whether LOPS of patients was prolonged or not. Variable continuity was interrupted by threshold-based classification, which could lead to information loss (35). In contrast, continuous variable prediction could fully retain original data information and accurately capture the inter-patient heterogeneity. In particular, continuous variable prediction could output quantitative values. Once the model training was completed, it could help focus attention on groups who actually need a longer LOPS and act as a tool to assist in medical decision-making when integrated into the current medical system.

In addition, hospital management need to strategically plan bed capacity and allocation to better match medical service providers with medical resources. Bed congestion could lead to resource waste, increased medical costs, and higher in-hospital risks for patients (36–38). On the other hand, bed vacancies might result in resource underutilization and financial losses (39). Hence, accurately predicting specific LOPS values is extremely important for hospital management. For example, the workload of nursing wards depended heavily on patient volume and their length of stay. The ability to forecast workload would enable managers to deliver cost-effective care. A cross-sectional study described the impact of nursing staff allocation on the length of hospital stay and hospitalization costs for patients undergoing hip or knee surgery (40). Yankovic et al. also described nursing staff allocation depend on the prediction of patients’ hospital stay duration, further supporting the model’s value for hospital management (41). Therefore, it was necessary to develop a prediction model for forecasting specific LOPS in elderly patients with hip fractures to optimize hospital management.

The BP-NN model could also offer an additional benefit, enabling patients to plan their schedules and medical expenses more systematically. Cao et al found that the LOPS of patients was 12 days (16), while another study indicated that the average LOPS for such patients was 16.6 days (31). In our study, the average LOPS of elderly patients with hip fractures was 17.42 days. Therefore, if the specific LOPS could be predicted based on the patients’ personal characteristics, rather than relying on empirical estimates, patients would be better able to coordinate life and medical-related affairs in advance. In terms of medical expenses, patients could also make reasonable plans and preparations to avoid the impact of cost issues on treatment. Although there was a large difference in LOPS among elderly patients with hip fractures between domestic and international settings due to factors such as post-discharge residence and healthcare systems, the prediction method proposed in this study was worthy of recommendation. Other regions could retrain the model on local datasets to attain clinically acceptable predictive performance. If necessary, hyperparameter optimization methods tailored to local data could be employed for iterative optimization, thereby realizing accurate LOPS prediction.

This study employed a BP-NN model to predict the specific LOPS in elderly patients with hip fractures based on EHR dataset. The model demonstrated robust performance in validation dataset (RMSE = 1.23 days, MAE = 1.57 days, MAPE = 7.69%), outperforming prior similar studies on continuous outcome prediction (31, 39). The coefficient of determination (*R*^2^ = 0.83) was moderately good, which was likely attributed to the inherent challenge with continuous outcome variable, including complexes conditions, interacting clinical factors and data noise. However, predicting LOPS as a categorize continuous might fail to reflect individual differences (42), since groups above or below the threshold were considered as inherently distinct. Critically, the relative error quantified by MAPE was of great significance for patient risk stratification and resource allocation. Nevertheless, RMSE, MAE, and MAPE used in this study had inherent limitations, with RMSE sensitive to extreme errors, MAE failing to distinguish error distributions, and MAPE tendance to exaggerate errors for short LOPS. Future studies could further incorporate indicators such as symmetric mean absolute percentage error and consistency correlation coefficient to measure precision and accuracy. Despite this fact, a specific LOPS prediction still provided a practical reference for individualized clinical decision-making.

SHAP analysis provided an interpretation of the LOPS prediction model, with age and ACCI being the main predictors, which were observed in previous retrospective analyses (16). With increasing age, elderly patients with hip fracture experienced varying degrees of decline in functional reserve, stress tolerance, and immunity, making them highly susceptible to various complications (43, 44), which may require an extended recovery period, thereby prolonging their LOPS. Sensitivity analysis with age treated as a categorical variable showed that the very old elderly group had the longest LOPS. This may be associated with factors such as relatively poor baseline health status and a higher burden of comorbidities in this population. Although age is an immutable clinical factor, this finding of the present study still holds important implications for the clinical risk management of specific patients and optimal allocation of medical resources. Cao et al. also reported that a high ACCI was associated with prolonged LOPS (16). ACCI cannot only serve as an indicator to evaluate the severity of preoperative diseases of patients (45) but also exhibit significant accuracy in predicting postoperative complications (46), and the severity of diseases and the incidence of complications largely affected patients’ LOPS (47). Although surgical type was a predictor of LOPS, there was no statistically significant difference in LOPS among the three surgical types after adjusting for confounding factors, suggesting that its independent effect on LOPS was modulated by age and ACCI. The specific mechanism underlying this result may be as follows: on the one hand, both age and ACCI were strongly correlated with LOPS in this study and served as the main dominant factors determining LOPS. On the other hand, the selection of surgical type in clinical practice was not an independent decision. Surgeons inherently made individualized decisions based on the patients’ age and health status, leading to a natural intertwining of the effects of surgical type with those of age and ACCI, and this ultimately results in the independent effect of surgical type being masked.

We interpret the potential limitations of this study in light of its findings. First, the retrospective design might introduce inherent selection and information biases. Although complete data of participants were obtained through standardized EHRs, there were still a few types of cases not included in this study. When patients with such transfer characteristics are encountered again, the model may have difficulty predicting their LOPS accurately. Second, this model was constructed solely based on data from elderly fracture patients in this region. However, LOPS may vary among patients in different countries, regions, and areas with varying economic levels. Therefore, it is necessary for other regions to develop models using local data to verify its generalizability. Third, this study collected only data from patients at admission based on EHRs to facilitate early intervention, while potential variables, such as frailty (48), preoperative medications (49), preoperative waiting time (admission-to-surgery interval), and American Society of Anesthesiologists’ (ASA) classification, were not included in model construction, which may limit the model’s performance. Fourth, this model was constructed based on the training dataset split in an 8:2 ratio. Although we supplemented 10-fold cross-validation to evaluate the model’s internal stability, no external validation has been conducted, and its external generalizability remains unclear. Future studies can perform multi-center external validation to clarify the model’s generalizability, and thereby enhance its clinical applicability. Fifth, this study constructed the LOPS prediction model only from the perspective of patient safety and did not incorporate objective socioeconomic factors such as income, medical expenses, and medical insurance status, so further research is needed to identify and overcome potential barriers in practical application.

## 5 Conclusion

This study developed and validated a BP -NN model using the EHRs of elderly patients with hip fractures to predict the specific LOPS in this population. The model identified age and ACCI as the strongest predictors, while also revealing that the impact of surgical type was not independent but was modulated through complex interactions with these patient-specific factors. These predictions indicate the potential to assist surgeons in making personalized decisions regarding the LOPS of elderly patients with hip fracture by considering the confluence of patient age, comorbidity, and planned surgical type, and in managing their in-hospital safety, as well as enable hospital administrators to more effectively plan medical resources strategically.

## Data Availability

The original contributions presented in this study are included in this article/supplementary material, further inquiries can be directed to the corresponding author.
